# Egr-1 increases angiogenesis in cartilage via binding Netrin-1 receptor DCC promoter

**DOI:** 10.1186/s13018-018-0826-x

**Published:** 2018-05-29

**Authors:** Jun Sheng, Da Liu, Xia Kang, Ying Chen, Kai Jiang, Wei Zheng

**Affiliations:** Department of Orthopedics, Chengdu Military General Hospital, 270 Rongdu Avenue, Jinniu District, Chengdu, 610083 Sichuan China

**Keywords:** Egr-1, Angiogenesis, Osteoarthritis, Netrin-1 receptor, DDC promoter

## Abstract

**Background:**

Osteoarthritis (OA) is a joint disease characterized by degradation of cartilage. The etiology of OA is still unclear. Vascular endothelial growth factor (VEGF) plays a key role of angiogenesis in the pathogenesis of OA and contributes to the angiogenesis of NT-1/DCC. Whether or not NT-1/DCC and VEGF interact in regulating angiogenesis of OA cartilage is not known.

**Methods:**

Histological studies for CD34, VEGF, and safranin-O staining were performed to determine angiogenesis and cartilage tissue injury. ELISA indicated the level of pro-inflammation cytokines. Immunoblotting, immunoprecipitation, and electrophoretic mobility shift assay (EMSA) were performed to assay the expression and function of NT-1/DCC-VEGF signaling pathway.

**Results:**

Our data indicated that VEGF expression was increased in cartilage tissue from OA rats, while the chondrocytes were disorganized, and cartilage degeneration was increasing in OA rats. The inflammation factors in articular cavity fluid were higher in the OA rats than in the sham. The protein expression of NT-1, DCC, and VEGF were increased in osteoarthritic cartilage. DCC was involved in the positive regulation of osteoarthritic angiogenesis by VEGF. Egr-1 expression was higher in OA rats than in sham rats. Egr-1 is a regulator of DCC promoter activity, and the binding is higher in OA rats than in sham rats.

**Conclusion:**

Our present study provides a mechanism by which Egr-1 induced angiogenesis via NT-1/DCC-VEGF pathway.

## Background

Osteoarthritis (OA) is an age-dependent, chronic, incurable, and destructive joint disease characterized by degradation of cartilage, hypertrophy of chondrocyte, and sclerosis of subchondral bone [1] and is a main cause of pain and disability of older individuals [2]. It seems that no pharmacological agents could prevent and treat OA, and alleviating joint pain could be the only medical option for OA, which is often unsuccessful, leading to total joint replacement [3, 4]. The lack of treatment may be ascribed to the unclear etiology of OA.

Articular cartilage is a highly specialized connective tissue with an avascular structure. However, cartilage loses the ability to stay avascular in the osteoarthritic environment [5], indicating that angiogenesis could contribute to the pathogenesis of OA. Previous researches found the contribution of angiogenesis of osteophyte formation in OA [6]. Angiogenesis is closely associated with the pathogenesis of OA [7]. Neovascularization modulates chondrocyte functions and contributes towards abnormal tissue growth and perfusion, ossification, and endochondral bone development [8] and leads to oxidative stress and inflammation, which results to matrix degradation [9]. Angiogenesis is regulated by a delicate balance between endogenous angiogenic and anti-angiogenic factors. It has been demonstrated that Netrin-1 (NT-1) is the factor regulating patterning of the vascular system [10], and NT-1 induced proliferation, migration, and tube formation of endothelial cells and human aortic smooth muscle cells, via Netrin-1 receptor DCC (Deleted in Colorectal Cancer) [11]. The vascular endothelial growth factor (VEGF) might contribute to the angiogenesis of NT-1/DCC [12]. VEGF plays a key role of angiogenesis in the pathogenesis of OA and is essential for establishing epiphyseal vascularization and remodeling hypertrophic cartilage, which finally leads to endochondral bone formation [13]. Whether or not NT-1/DCC and VEGF interact in regulating angiogenesis of OA cartilage is not known. And the upstream signaling that regulates NT-1/DCC-VEGF to angiogenesis in cartilage is still unclear.

Early growth response-1 (Egr-1), also called NGFI-A, is a zinc finger transcription factor and immediate early gene, which plays an important role in angiogenesis [14, 15]. Meanwhile, Egr-1 may be involved in the pathogenesis of OA [16, 17]. Few studies report the effect of Egr-1 to angiogenesis in osteoarthritic cartilage. Thus, our present study provides a mechanism by which Egr-1 induced angiogenesis via NT-1/DCC-VEGF pathway.

## Methods

### Animals

Lewis rats (260–280 g) were purchased from Chengdu Da Shuo Biotech Co. Ltd. (Chengdu, China) and randomized to the sham group and OA group, *n* = 8 per group. The rat OA model was performed as anterior cruciate ligament transection (ACLT). The ACLT was conducted as described in the previous study [18], which induced mechanical instability-associated OA. The rats were euthanized 2 months after ACLT. And siRNA or recombinant netrin 1 were injected into knee joints 48 h before euthanasia. The limbs of OA rats were dissected for staining, and articular cavity fluid were collected for further research.

All experiments conformed to the guidelines of the ethical use of animals, and all efforts were made to minimize animal suffering and to reduce the number of animals used.

### Histological study

Cartilage tissues from rats in each group were fixed with 4% paraformaldehyde for 24 h and decalcified for 2 weeks with 20% EDTA at 4 °C. After embedding in paraffin, the tissues were sectioned to 4-μm slices and mounted on slides. The slides were incubated with anti-CD34 (1:100) for determining microvessel density (MVD) or anti-VEGF (1:100) antibody (Santa Cruz Biotechnology, CA) overnight at 4 °C followed by immunofluorescence and immunohistochemistry, or stained with 0.1% safranin-O for 30 min (Sigma Aldrich, St. Louis, MN). To assess microvessel density in epiphysis tissues, the number of new blood vessels visualized by CD34^+^ vascular endothelial cells was counted per high-power field (× 400) [19].

### ELISA and immunoblotting

The concentration of IL-1β and TNF-α in articular cavity fluid was determined by the ELISA kit (R&D Systems) according to the manufacturer’s instructions. And tissues were washed three times with PBS, pulverized in liquid nitrogen, and lysed [20]. The homogenates (20 μg of protein) were separated by 8% SDS-PAGE and blotted on polyvinylidene fluori (PVDF) membranes (Bio–Rad Laboratories). Transblots were probed with the rabbit anti-Egr-1 (Cell Signaling technology Inc., 1:1000), rabbit anti-VEGF antibody (Santa Cruz Biotechnology, 1:400), rabbit anti-DCC antibody (Santa Cruz Biotechnology, 1:400), or rabbit anti-Netrin-1 antibody (Santa Cruz Biotechnology, 1:400) to examine the protein expression in the lysates. The amount of protein transferred onto the membranes was verified by immunoblotting for GAPDH (Santa Cruz Biotechnology, 1:500).

### Immunoprecipitation

Equal amounts of homogenates (400 μg of protein) were incubated with affinity-purified anti-DCC antibody for 1 h and protein-G agarose at 4 °C for 12 h. The immunoprecipitates were suspended in a sample buffer and subjected to immunoblotting with the VEGF antibody. To determine the specificity of the bands found on the immunoblots, IgG (negative control) and anti-DCC antibody (positive control) were used as the immunoprecipitants.

### Small interfering RNA

Small interfering RNA (siRNA) against rat DCC (NM_012841) and Egr-1 (NM_012551) mRNA was synthesized and purified by Qiagen (Hilden, Germany). The effects of 50-nM siRNA were compared with scrambled RNA (negative control, Qiagen). Briefly, 10-nM siRNA or control RNA were mixed with 6 μL of oligofectamine in Optimem medium (Invitrogen Life Technologies) and incubated with cells grown in six-well plates for 24 h and then switched to growth medium and incubated for another 24 h.

### EMSA

Electrophoretic mobility shift assays (EMSAs) were performed as described before [21, 22], by the Light-shift Chemiluminescent EMSA Kit (Pierce Chemical Co., Rockford, IL). A synthetic DNA double-stranded oligonucleotide probe (5′-biotin-CGGTACATGACACAGGCTGAC-5′) containing the sequence of the rat DCC gene promoter between nucleotides -101 and -91 bp (5′-CCAGCTCGCA-3′) was labeled with biotin and incubated with the nuclear extracts. Rabbit anti-Egr-1 (Cell Signaling technology Inc.) was used for supershift experiments. A chemiluminescent detection method with a luminal/enhancer solution and stable peroxide solution (Pierce Chemical Co., Rockford, IL) was used, as described by the manufacturer, and the membranes were exposed to X-ray films for 30 s to 5 min before development.

### Statistical analysis

The data are expressed as mean ± SEM. Comparison within groups was made by *t* test or one-way ANOVA for repeated measures. A value of *P* < 0.05 was considered significant.

## Results

### Angiogenesis in cartilage of OA rat

**Fig. 1:**
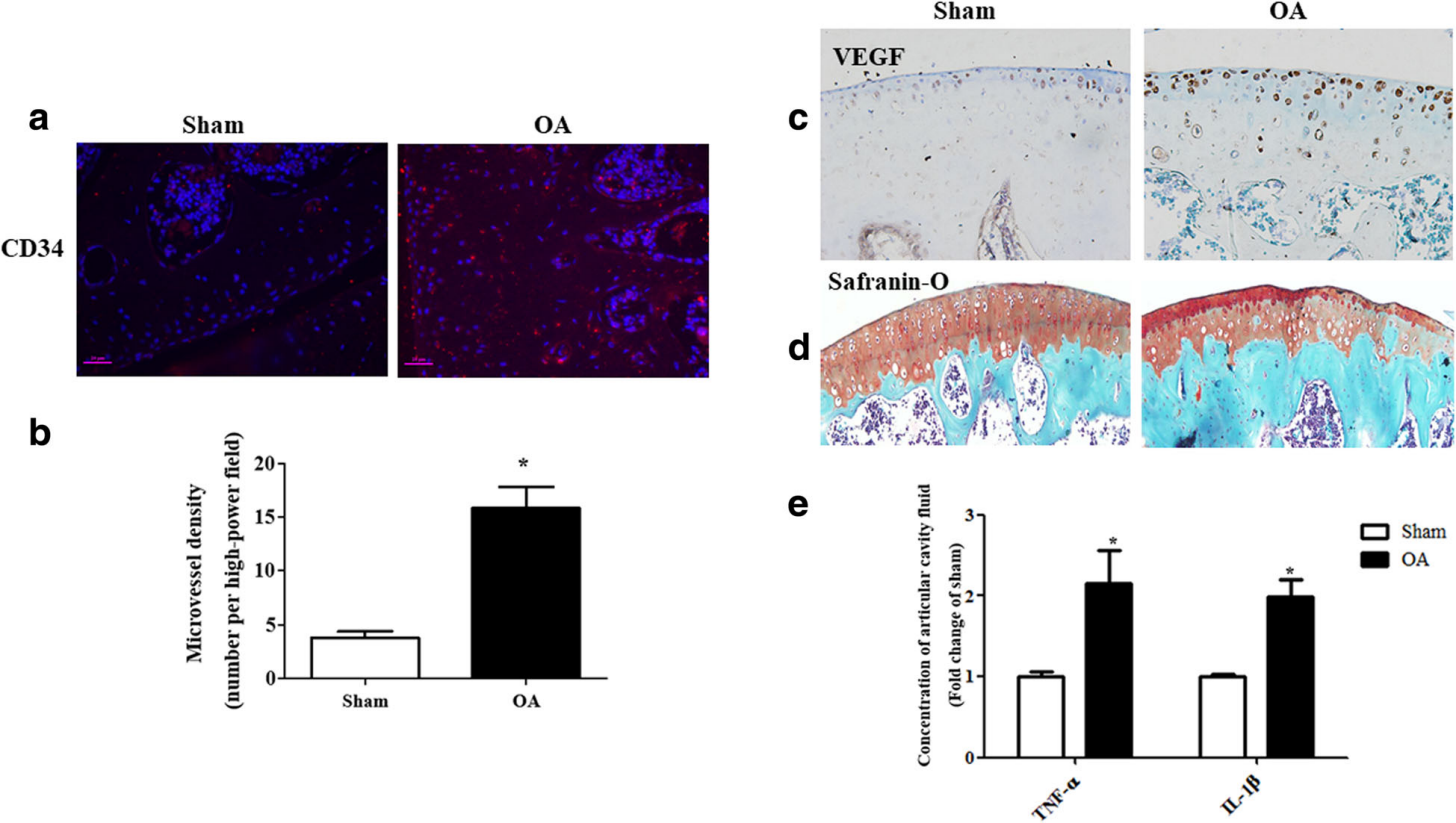
Angiogenesis in cartilage of OA rat. **a**, **b** Immunofluorescence staining of CD34. CD34 immunofluorescence showed the stained microvessels. The number of new blood vessels visualized by CD34^+^ vascular endothelial cells was counted per high-power field (× 400). (*P = 0.0004 vs. sham, *n* = 5, scale bar = 20 μm). **c**, **d** Immunohistochemical staining of VEGF. The severity of proteoglycan depletion in OA cartilage was demonstrated by safranin-O staining. **e** The levels of IL-1β and TNF-α were measured by ELISA (*P = 0.0001 vs. others, *n* = 5)

To check the angiogenesis in OA rat, the microvessel density in epiphysis tissues of the proximal epiphysis of the tibia was quantified via CD34 staining, and found microvessel density significantly increased in OA cartilage tissue (Fig. 1a, b). The data showed that VEGF expression was increased in cartilage tissue from OA rats (Fig. 1c), indicating that proliferation and migration of endothelial cell were promoted in OA. Moreover, the chondrocytes were disorganized, and cartilage degeneration was increasing in OA rats (Fig. 1d). The TNF-α and IL-1β levels in articular cavity fluid were higher in the OA rats than in the sham (Fig. 1e). These data suggest that angiogenesis could contribute to the pathogenesis of OA.

### DCC regulates the angiogenesis in OA via VEGF

**Fig. 2:**
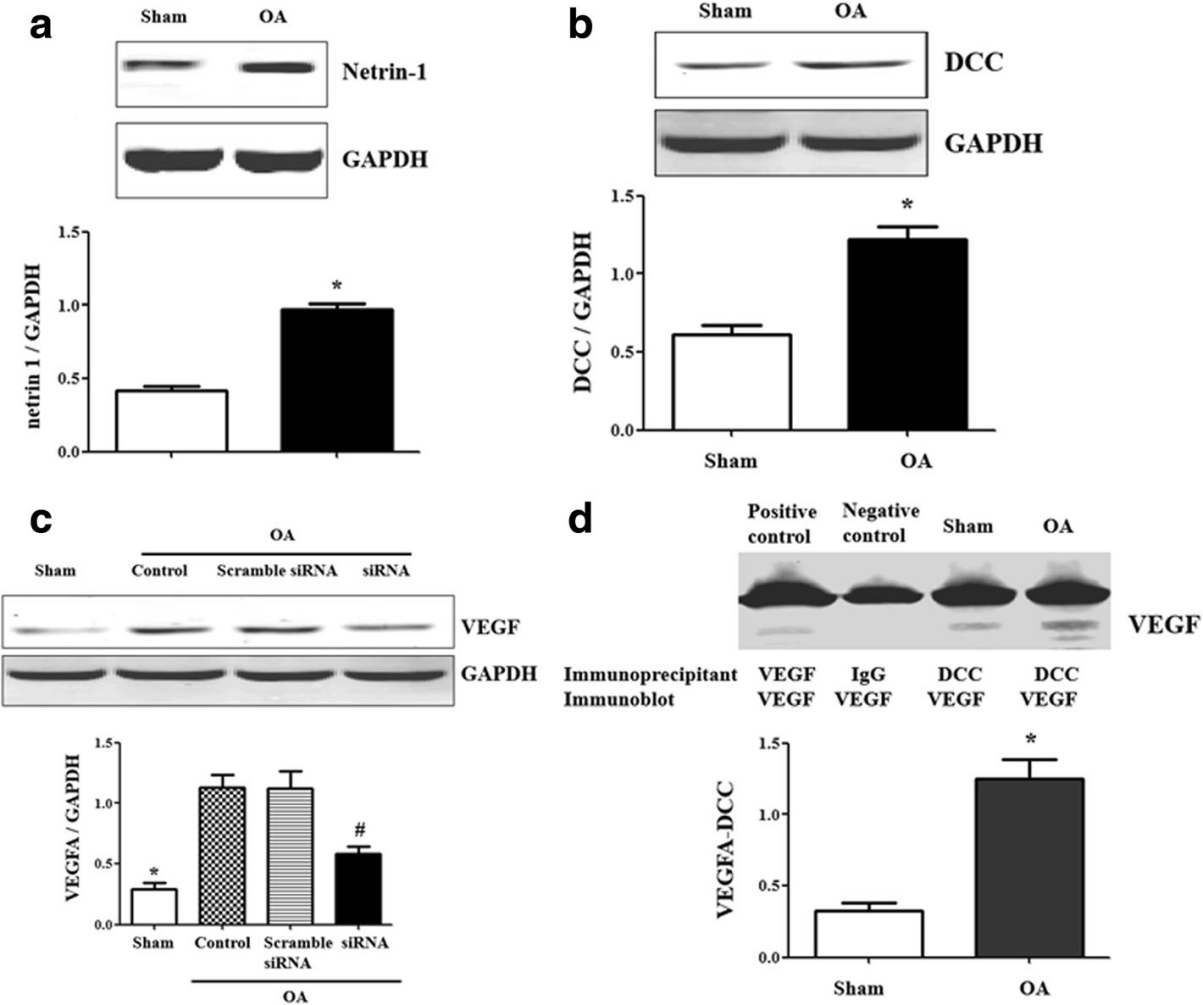
DCC regulates the angiogenesis in OA via VEGF. **a** Netrin-1 expression in OA cartilage. Results are expressed as the ratio of Netrin-1 and GAPDH (*P = 0.00009 vs. the sham, *n* = 5 in each group). **b** DCC expression in OA cartilage. Results are expressed as the ratio of DCC and GAPDH (*P = 0.0012 vs. the sham, *n* = 5 in each group). **c** VEGF expression in DCC siRNA-treated OA cartilage. Results are expressed as the ratio of VEGF and GAPDH (*P = 0.0002 vs. the others, ^#^*P* = 0.0003 vs. OA group and scramble siRNA-treated group, *n* = 5 in each group). **d** Co-immunoprecipitation of DCC and VEGF in OA cartilage. The lysates were immunoprecipitated with DCC antibody and immunoblotted with VEGF antibodies (*P = 0.0008 vs. sham, *n* = 4, lane 1 = positive control, lane 2 = negative control, lane 3 = sham, lane 4 = OA group. For the positive control, VEGF antibody was used, and for the negative control, IgG was used instead of DCC antibody as the immunoprecipitants)

Previous study demonstrated that NT-1 and its receptor, DCC, stimulate growth of umbilical vein endothelial cells and vascular smooth muscle [23]. However, the role of NT-1/DCC in angiogenesis of osteoarthritic cartilage is still unclear. Thus, our studies checked the expression of NT-1 and DCC in cartilage and found that the protein expression of NT-1 and DCC were increased in osteoarthritic cartilage (Fig. 2a, b).

Besides, VEGF protein expression was significantly higher in the OA rats than in the sham, while the DCC siRNA decreased the increasing VEGF expression (Fig. 2c), indicating DCC was involved in the positive regulation of osteoarthritic angiogenesis by VEGF.

An additional study found a co-immunoprecipitation (Fig. 2d) between DCC and VEGF; the co-immunoprecipitation of DCC and VEGF was high in OA rats than in sham rats, which could be a factor in the increased expression of VEGF in OA rats.

### The binding of Egr-1 at DCC promoter regulates the angiogenesis

**Fig. 3:**
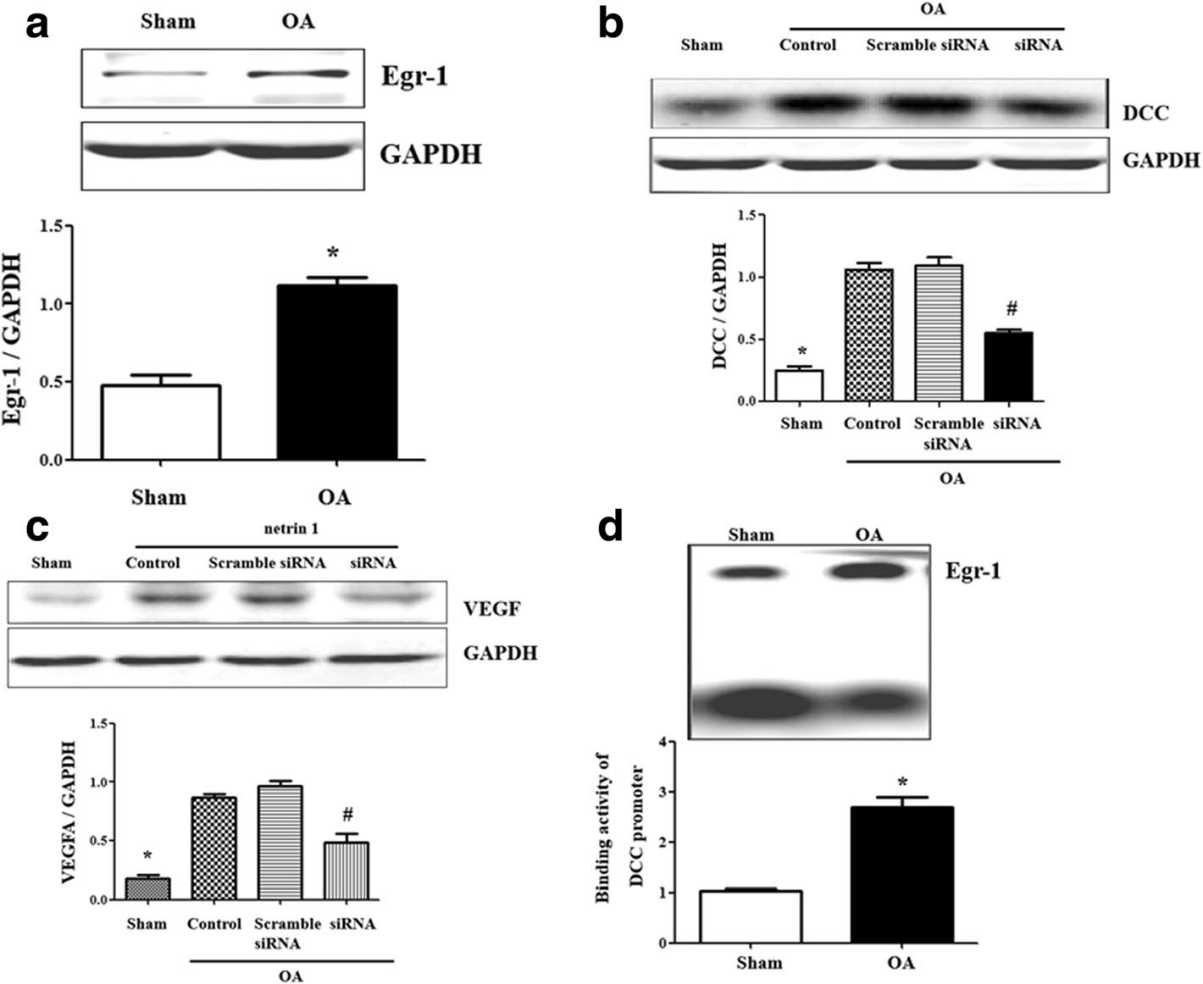
The binding of Egr-1 at DCC promoter regulates the angiogenesis. **a** Egr-1 expression in OA cartilage. Results are expressed as the ratio of Egr-1 and GAPDH (*P = 0.0003 vs. the sham, *n* = 5 in each group). **b** DCC expression in Egr-1 siRNA-treated OA cartilage. Results are expressed as the ratio of DCC and GAPDH (*P = 0.0001 vs. the others, ^#^*P* = 0.0005 vs. OA group and scramble siRNA-treated group, *n* = 5 in each group). **c** VEGF expression in Netrin-1 and Egr-1 siRNA-treated cartilage. Results are expressed as the ratio of VEGF and GAPDH (*P = 0.00005 vs. the others, ^#^*P* = 0.00009 vs. OA group and scramble siRNA-treated group, *n* = 5 in each group). **d** EMSA of nuclear protein from cartilage tissue. Binding activity of DCC gene promoter, containing an Egr-1 site, was examined in nuclear protein (*P = 0.0002 vs. the sham, *n* = 5 in each group)

To investigate whether or not Egr-1 was involved in the regulation of DCC on angiogenesis, we measured the Egr-1 expression in cartilage firstly. Egr-1 protein expression was higher in OA rats than in sham rats (Fig. 3a). The Egr-1 siRNA decreased the increasing DCC expression in osteoarthritic cartilage (Fig. 3b) and decreased the NT-1-induced VEGF expression (Fig. 3c).

To identify the hypothesis that Egr-1 is a regulator of DCC promoter activity, we measured Egr-1 binding to the DCC promoter and found it higher in OA rats than in sham rats (Fig. 3d), indicating that the regulation of DCC by Egr-1 occurred at transcriptional level.

## Discussion

The balance between angiogenic and anti-angiogenic factors is an important regulator of cartilage homeostasis [24–26]. And healthy adult human articular cartilage is avascular, and blood vessels could penetrate newly formed cartilage at the joint margins in OA and increase osteophyte formation, which may contribute to symptoms and joint damage [5, 27, 28]. In the present study, we found that the endothelial cell proliferation, vascular density, and VEGF abundance were increased in osteoarthritic cartilage which lead to the cartilage degeneration. Besides the stimulation of angiogenesis, VEGF may also contribute to inflammation [29, 30]. Indeed, our data show a significant upregulation in osteoarthritic articular cavity fluid of cytokines such as TNF-α and IL-1β. The pro-inflammatory factors are the major mediators of the catabolism of OA cartilage and stimulator for osteoarthritic chondrocyte [31–33]. Thus, VEGF seems to induce osteoarthritis via angiogenesis and inflammatory. And whether or not NT-1/DCC and VEGF interact in regulating angiogenesis of OA cartilage is not known.

Further studies identified the increasing expression of NT-1/DCC in osteoarthritic cartilage and increasing co-immunoprecipitation between DCC and VEGF. Meanwhile, the DCC siRNA decreased the increasing VEGF expression in OA. These data indicated DCC was involved in the positive regulation of osteoarthritic angiogenesis by VEGF directly in OA. The mechanisms of DCC in the regulation of VEGF are still unclear. Previous studies found that DCC, on NT-1 binding, activates the extracelluar signal-regulated kinase-1/2 (ERK-1/2) and mitogen-activated protein kinase (MAPK) [34, 35]. And DCC was also shown to activate the ERK-1/2-eNOS pathway [36]. It was reported earlier that NO mediates VEGF-induced angiogenesis [37]. MAPK/ERK signaling pathway also plays an important role in the regulation of VEGF [38, 39]. We infer that the DCC could upregulate VEGF via MAPK/ERK or eNOS/NO signaling pathway. However, more data from further studies are necessary to support the hypothesis.

As an active transcription factor, Egr-1 regulates promoter activity of various proteins. We believe the Egr-1 could be an upstream regulator of DCC/VEGF pathway. The expression of Egr-1 in OA cartilage has been controversial. Despite Wang et al. found the Egr-1 expression in OA cartilage is decreased [40], we demonstrated that Egr-1 protein expression was higher in OA rats than in sham rats. This is consistent with previous studies showing that Egr-1 expression increases in OA cartilage [17]. The Egr-1-mediated regulation of DCC expression might be complicated, as in our present study, we found that Egr-1 siRNA decreased the increasing DCC expression in osteoarthritic cartilage and NT-1-induced VEGF expression. And our data uncover a possible mechanism: The activity of Egr-1, a regulator of DCC promoter activity, is increased, accompanied by an increase in its binding to the DCC promoter in OA cartilage. And the pathway leading to the higher binding of Egr-1 with DCC promoter is not known, which needs to be elucidated in the future.

The current study has several limitations. First, chromatin immunoprecitation (ChIP) may be used to identify the increased binding of Egr-1 to the DCC promoter in OA cartilage, rather than EMSA. Second, while our present study focused on the role of Egr-1/DCC pathway, whether or not Egr-1 or DCC could be a therapy target is another topic of future study.

## Conclusion

In conclusion, the present study reinforces the role of Egr-1 in OA and shows that an increased expression of Egr-1 increases cartilage DCC expression, which may be involved in the abnormalities of angiogenesis in OA. The results imply that Egr-1 may be an effective therapeutic target for OA.

## Abbreviations

ACLT: Anterior cruciate ligament transection
DCC: Deleted in Colorectal Cancer
Egr-1: Early growth response-1
ERK-1/2: Extracelluar signal-regulated kinase-1/2
MAPK: Mitogen-activated protein kinase
MVD: Determining microvessel density
NT-1: Netrin-1
OA: Osteoarthritis
PVDF: Polyvinylidene fluori
VEGF: Vascular endothelial growth factor

## References

[1] Neogi T, Zhang Y (2013). Epidemiology of osteoarthritis. Rheum Dis Clin N Am.

[2] Vina ER, Ran D, Ashbeck EL, et al. Natural history of pain and disability among African-Americans and Whites with or at risk for knee osteoarthritis: a longitudinal study. Osteoarthr Cartil. 2018; 10.1016/j.joca.2018.01.020. 10.1016/j.joca.2018.01.020 PMC5871565 29408279

[3] Steinberg J, Zeggini E (2016). Functional genomics in osteoarthritis: past, present, and future. J Orthop Res.

[4] Tiku ML, Sabaawy HE (2015). Cartilage regeneration for treatment of osteoarthritis: a paradigm for nonsurgical intervention. Ther Adv Musculoskelet Dis.

[5] Walsh DA, McWilliams DF, Turley MJ (2010). Angiogenesis and nerve growth factor at the osteochondral junction in rheumatoid arthritis and osteoarthritis. Rheumatology (Oxford).

[6] Mapp PI, Walsh DA (2012). Mechanisms and targets of angiogenesis and nerve growth in osteoarthritis. Nat Rev Rheumatol.

[7] Zhao C, Liu Q, Wang K (2017). Artesunate attenuates ACLT-induced osteoarthritis by suppressing osteoclastogenesis and aberrant angiogenesis. Biomed Pharmacother.

[8] Kobayashi T, Kakizaki I, Nozaka H (2017). Chondroitin sulfate proteoglycans from salmon nasal cartilage inhibit angiogenesis. Biochem Biophys Rep.

[9] Noh KC, Park SH, Yang CJ (2018). Involvement of synovial matrix degradation and angiogenesis in oxidative stress-exposed degenerative rotator cuff tears with osteoarthritis. J Shoulder Elb Surg.

[10] Chen J, Du H, Zhang Y (2017). Netrin-1 prevents rat primary cortical neurons from apoptosis via the DCC/ERK pathway. Front Cell Neurosci.

[11] Lu H, Wang Y, He X (2012). Netrin-1 hyperexpression in mouse brain promotes angiogenesis and long-term neurological recovery after transient focal ischemia. Stroke.

[12] Yu Y, Zou J, Han Y (2015). Effects of intravitreal injection of netrin-1 in retinal neovascularization of streptozotocin-induced diabetic rats. Drug Des Devel Ther.

[13] Nagao M, Hamilton JL, Kc R (2017). Vascular endothelial growth factor in cartilage development and osteoarthritis. Sci Rep.

[14] Wang LF, Liu YS, Yang B (2018). The extracellular matrix protein mindin attenuates colon cancer progression by blocking angiogenesis via Egr-1-mediated regulation. Oncogene.

[15] Yoon YJ, Kim DK, Yoon CM (2014). Egr-1 activation by cancer-derived extracellular vesicles promotes endothelial cell migration via ERK1/2 and JNK signaling pathways. PLoS One.

[16] Rockel JS, Bernier SM, Leask A (2009). Egr-1 inhibits the expression of extracellular matrix genes in chondrocytes by TNFalpha-induced MEK/ERK signalling. Arthritis Res Ther.

[17] Nebbaki SS, El Mansouri FE, Afif H (2012). Egr-1 contributes to IL-1-mediated down-regulation of peroxisome proliferator-activated receptor gamma expression in human osteoarthritic chondrocytes. Arthritis Res Ther.

[18] Tsai HC, Chen TL, Chen YP, et al. Traumatic osteoarthritis-induced persistent mechanical hyperalgesia in a rat model of anterior cruciate ligament transection plus a medial meniscectomy. J Pain Res. 10.2147/JPR.S154038. 10.2147/JPR.S154038 PMC5743113 29317848

[19] Yigit N, Covey S, Barouk-Fox S (2015). Nuclear factor-erythroid 2, nerve growth factor receptor, and CD34-microvessel density are differentially expressed in primary myelofibrosis, polycythemia vera, and essential thrombocythemia. Hum Pathol.

[20] Tajima T, Sekimoto T, Yamaguchi N (2017). Hemoglobin stimulates the expression of ADAMTS-5 and ADAMTS-9 by synovial cells: a possible cause of articular cartilage damage after intra-articular hemorrhage. BMC Musculoskelet Disord.

[21] Ellmann L, Joshi MB, Resink TJ (2012). BRN2 is a transcriptional repressor of CDH13 (T-cadherin) in melanoma cells. Lab Investig.

[22] Ruedel A, Stark K, Kaufmann S (2014). N-cadherin promoter polymorphisms and risk of osteoarthritis. FASEB J.

[23] Park KW, Crouse D, Lee M (2004). The axonal attractant Netrin-1 is an angiogenic factor. Proc Natl Acad Sci U S A.

[24] Haywood L, McWilliams DF, Pearson CI (2003). Inflammation and angiogenesis in osteoarthritis. Arthritis Rheum.

[25] Bonnet CS, Walsh DA (2005). Osteoarthritis, angiogenesis and inflammation. Rheumatology (Oxford).

[26] Ashraf S, Mapp PI, Walsh DA (2011). Contributions of angiogenesis to inflammation, joint damage, and pain in a rat model of osteoarthritis. Arthritis Rheum.

[27] Suri S, Gill SE, Massena de Camin S (2007). Neurovascular invasion at the osteochondral junction and in osteophytes in osteoarthritis. Ann Rheum Dis.

[28] Ashraf S, Walsh DA (2008). Angiogenesis in osteoarthritis. Curr Opin Rheumatol.

[29] Ferrara N, Gerber HP (2001). The role of vascular endothelial growth factor in angiogenesis. Acta Haematol.

[30] Ferrara N (2001). Role of vascular endothelial growth factor in regulation of physiological angiogenesis. Am J Physiol Cell Physiol.

[31] Kapoor M, Martel-Pelletier J, Lajeunesse D (2011). Role of proinflammatory cytokines in the pathophysiology of osteoarthritis. Nat Rev Rheumatol.

[32] Genemaras AA, Ennis H, Kaplan L (2016). Inflammatory cytokines induce specific time- and concentration-dependent MicroRNA release by chondrocytes, synoviocytes, and meniscus cells. J Orthop Res.

[33] Zhao YP, Liu B, Tian QY (2015). Progranulin protects against osteoarthritis through interacting with TNF-alpha and beta-Catenin signalling. Ann Rheum Dis.

[34] Forcet C, Stein E, Pays L (2002). Netrin-1-mediated axon outgrowth requires deleted in colorectal cancer-dependent MAPK activation. Nature.

[35] Lange J, Yafai Y, Noack A (2012). The axon guidance molecule Netrin-4 is expressed by Muller cells and contributes to angiogenesis in the retina. Glia.

[36] Nguyen A, Cai H (2006). Netrin-1 induces angiogenesis via a DCC-dependent ERK1/2-eNOS feed-forward mechanism. Proc Natl Acad Sci U S A.

[37] Papapetropoulos A, Garcia-Cardena G, Madri JA (1997). Nitric oxide production contributes to the angiogenic properties of vascular endothelial growth factor in human endothelial cells. J Clin Invest.

[38] Chen J, Gu Z, Wu M (2016). C-reactive protein can upregulate VEGF expression to promote ADSC-induced angiogenesis by activating HIF-1alpha via CD64/PI3k/Akt and MAPK/ERK signaling pathways. Stem Cell Res Ther.

[39] Zhang L, Zhang ZK, Liang S. Epigallocatechin-3-gallate protects retinal vascular endothelial cells from high glucose stress in vitro via the MAPK/ERK-VEGF pathway. Genet Mol Res. 2016;15(2) 10.4238/gmr.15027874. Epub 2016/06/21 10.4238/gmr.15027874 27323164

[40] Wang FL, Connor JR, Dodds RA, James IE, Kumar S, Zou C (2000). Differential expression of egr-1 in osteoarthritic compared to normal adult human articular cartilage. Osteoarthr Cartil.

